# The Effect of Fat Supplementation on the Appearance of Symptoms Associated With Dumping Syndrome in Patients Having Undergone Gastric Surgery: Preliminary Results

**DOI:** 10.7759/cureus.48871

**Published:** 2023-11-15

**Authors:** Athanasios Migdanis, Georgios D Koukoulis, Dimitrios Chougias, Ioannis Migdanis, Eleni Armeni, Spyridon Kanellakis, Athanasios Manouras, Andreas Kapsoritakis, Spyridon Potamianos

**Affiliations:** 1 Faculty of Medicine, University of Thessaly, Larissa, GRC; 2 Department of Nutrition and Dietetics, University of Thessaly, Trikala, GRC; 3 Department of General Surgery, General Hospital of Larissa, Larissa, GRC; 4 Department of Gastroenterology, General University Hospital of Larissa, Larissa, GRC; 5 Department of Nutrition and Dietetics, Harokopio University of Athens, Athens, GRC

**Keywords:** ds symptoms, glycaemic response, fat supplementation, gastric surgery, dumping syndrome

## Abstract

Background/Objectives: Data on the effect of dietary fat on dumping syndrome (DS) symptoms are limited. The aim of this study was to assess the effect of the addition of fat to a carbohydrate meal on the appearance of DS symptoms and glycemic response, in patients who had undergone gastric surgery.

Subjects/Methods: This was an interventional crossover study. Patients scheduled for gastric surgical procedures related to DS at two surgical units of two public hospitals (General University Hospital of Larissa and General Hospital of Larissa) were considered for study inclusion. Patients presenting symptoms suggestive of diagnosis (n = 12), after the ingestion of a carbohydrate meal, were used as both intervention and control groups. During the intervention process, a fat supplement was added to the carbohydrate meal that was previously used for diagnosis. Glycemic response and the amount and intensity of DS symptoms provoked by the two meals were assessed at both appointments.

Results: Blood glucose levels were significantly lower in the group that consumed the added fat meal compared with the group that consumed the carbohydrate meal 60 minutes after ingestion (p = 0.028). Furthermore, a significant reduction was noted in the amount of late dumping symptoms (p = 0.021) and the intensity of both early and late dumping symptoms (p = 0.007 and p = 0.012 respectively), after fat addition.

Conclusions: Incorporating fat into a carbohydrate meal seems to attenuate postprandial blood glucose rises and reduce the amount and intensity of DS symptoms, in patients who had undergone gastric surgery.

## Introduction

A possible adverse effect of cancer and non-cancer esophageal and gastric surgery as well as bariatric surgery is dumping syndrome (DS) [1]. According to several scientific data, DS has been reported to occur in patients who undergo vagotomy with pyloroplasty (approximately 20%), in patients after bariatric surgical procedures including Roux-en-Y gastric bypass (RYBP) or sleeve gastrectomy (25-50%) and in patients who undergo esophagectomy (up to 50%) [1-7]. Regarding gastrectomy for gastric cancer surgery, DS is also common with an incidence rate varying between 27% and 79% depending on the procedure [8]. DS is also seen after Nissen fundoplication in children and adults [9-12]. This syndrome is distinguished into two types according to the symptoms it causes postprandially. The majority of patients have early dumping presenting symptoms such as bloating and diarrhea 15 to 30 minutes after eating a meal, approximately 25% of them have late dumping developing hypoglycemia 1-3 hours postprandially, and only a minority have symptoms of both [13].

The diagnosis of the syndrome occurs postoperatively, usually following a patient's complaint of annoyances not related to complications of the operation. Diagnosis of DS is quite difficult, as some of the symptoms of the condition appear in other diseases or post-operative complications. The fact is that DS diagnosis comes after exclusion of all other diseases due to their similarity and possible misinterpretation. Various approaches can be used to detect clinically meaningful symptoms and confirm diagnosis, including symptom-based questionnaires (Sigstad’s score and Art’s dumping questionnaire) [14,15], oral glucose challenge testing, glycemia measurements, and gastric emptying studies [2,16].

Treatment options for DS consist of dietary modifications, pharmacologic interventions and possibly, as a last resort, surgical restoration of the stomach anatomy. The nutritional management of the syndrome involves avoiding consumption of simple carbohydrates (sweets, sugar, fruit juices, etc.) and liquids during meals. Changing dietary behavior and encouraging patients to consume small and frequent meals seem to have positive results [3,16]. Moreover, increasing dietary fiber can help treat hypoglycemia by slowing gastric emptying. Proteins also elicit fewer symptoms and should be increased, as well as fats. This helps alleviate symptoms, as well as provide sufficient caloric intake to prevent malnutrition (due to restricted intake of carbohydrates or fear of eating) [2,13].

Scientific data on the effect of dietary fat intake on DS symptoms are sparse. The in vivo effect of fat on gastric emptying and glycemic response has been investigated by several studies in non-DS patients, and data show that addition of fat to a carbohydrate meal can slow gastric emptying and attenuate postprandial rises in glucose [17-19]. On the contrary, a study that assessed the effect of fat on dumping symptoms showed that a meal with predominant fat content resulted in as many perceived dumping symptoms as a carbohydrate-profiled meal [20].

The aim of the present study was to assess the effect of the addition of fat to a carbohydrate meal on the appearance of DS symptoms and glycemic response, in patients who had undergone gastric surgery. 

## Materials and methods

Patients were included if they were adults and had undergone elective surgical procedures related to DS. Εxclusion criteria were type I and II diabetes mellitus and patients treated with corticosteroids, as both can potentially affect blood glucose levels. Furthermore, patients with medical history of gastrointestinal disorders (e.g. irritable bowel syndrome, Crohn’s disease, ulcerative colitis, etc) or history of previous bowel resections were also excluded from the study, as the above conditions could affect the symptoms related to DS. Sample collection was carried out in the Gastroenterology and Surgical Unit of General University Hospital of Larissa and the Surgical Unit of General Hospital of Larissa, Greece. Informed consent was obtained from all patients before entering the study. The trial protocol was approved by the Hospital’s Ethics Committee and adhered at all times to the Helsinki Declaration. The study was also registered at one of the available official sites for clinical trials registration (ClinicalTrials.gov ID: NCT05759689).

The study was initiated on April 2022 and is in progress. A total of 40 patients consented and were assessed for DS; 14 of which presented symptoms suggestive of diagnosis (during the first appointment; after the ingestion of a high carbohydrate liquid meal) according to Sigstad’s score (> 7) and were recruited for the study. During the intervention process, two patients dropped out as they could not tolerate the taste of the oral supplements used, leaving 12 patients for analysis. All patients who participated in the study were at 6-18 months post-surgery and had undergone subtotal gastrectomy with Roux-en Y reconstruction for gastric cancer or sleeve gastrectomy for obesity. 

Intervention process

Patients arrived in the outpatient clinic of the hospital on two different mornings after overnight fasting. The participants consumed a low-fat carbohydrate drink (diagnosis appointment) and a fat-added carbohydrate drink (intervention appointment) on two different days in a crossover pattern, thus requiring two study visits. Blood glucose levels were measured via a saccharometer before both liquid test meals and at 30, 60, 90, and 120 minutes after finishing the meal. At each appointment, at hours 1 and 2 after the consumption of the test meal, Sigstad’s score and Art’s dumping questionnaire were both used to assess symptoms relevant to DS. Sigstad’s scoring system [14] assigns points to each dumping symptom (e.g. dizziness, nausea, abdominal fullness), and total points are used to calculate a diagnostic index. A total score of >7 is considered diagnostic of DS, whereas a score of <4 suggests that other diagnosis should be considered. The Art’s dumping score [15] was used to assess the severity of symptoms one hour after the ingestion of the test meals for early dumping, and at two hours for late dumping. This is a score developed based on standard early or late dumping symptoms (eight and six symptoms, respectively), the intensity of which is described using a four-point Likert scale, where 0 represents the absence of symptoms, 1 mild, 2 relevant, and 3 severe intensity.

The test meal used at the first appointment was a high carbohydrate oral meal (Resource Fruit; Nestle Nutrition) which contained 254 kcal, 54g carbohydrates, 8g proteins, and 0g fat in 200ml. During the second appointment, the same oral supplement was administered in combination with 30ml of Calogen (Nutricia Medical Nutrition) which contained 135 kcal, 0g carbohydrates, 0g proteins, and 15g fat. 

Statistical analysis

Statistical analysis was conducted using IBM SPSS Statistics for Windows, Version 26 (Released 2019; IBM Corp., Armonk, New York, United States). Continuous variables such as age, anthropometric characteristics, biochemical parameters, and symptom-based questionnaire scores are presented as means and ± standard deviations. Comparison of continuous variables (glucose levels, Sigstad’s and Art’s score) among the two designated time points (1st appointment; assessment without the fat supplement and 2nd appointment; assessment with the fat supplement) was carried out using the non-parametric Wilcoxon signed-rank test. Statistical significance is reported as p < 0.05. 

## Results

**Figure 1:**
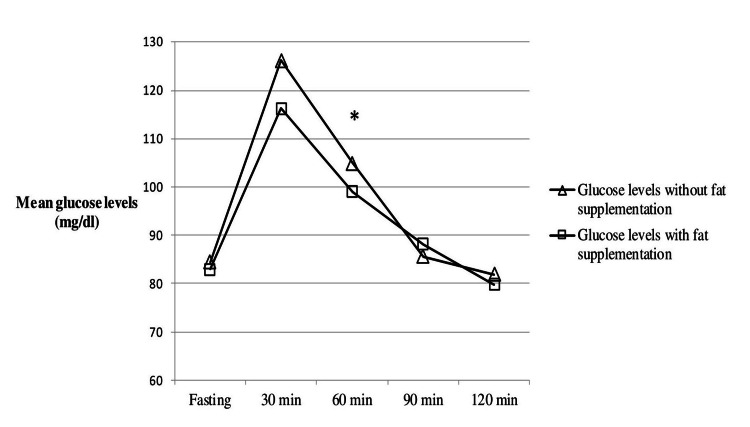
Differences in mean glucose levels between the group that consumed the carbohydrate meal and the group that consumed the fat-added carbohydrate meal. * indicates significant differences in mean glucose values between the two groups (p < 0.05).

Statistical analysis was performed on a final sample of five male and seven female patients. The mean age and BMI of the participants was 55±14.3 years and 30.7±6.4 kg/m^2^ respectively (Table 1). Glycemic response of the subjects was generally reduced after fat addition during the second appointment. Particularly, mean blood glucose levels were significantly lower in the group that consumed the added fat test meal (99±7 mg/dl) compared with the group that consumed the carbohydrate test meal (104.9±7 mg/dl) at 60 minutes after the ingestion of the meals (p = 0.028). No statistical differences were observed in measured glucose mean values at any other time point (0, 30, 90, 120 minutes) between the two groups of the study (p > 0.05) (Table 2). Furthermore, the data presented in Figure 1 demonstrate the mean glycemic response, after the consumption of the carbohydrate test meal compared to the consumption of the fat-added meal. 

**Table 1:** Demographic and anthropometric characteristics of the participants BMI, body mass index; SD, standard deviation

Variables	Participants (mean±SD/number of cases) n = 12
Gender	
Males	5
Females	7
Time post operation (months)	10±3.7
Age (years)	55±14.3
Weight (kg)	89±16.7
BMI (kg/m^2^)	30.7±6.4

**Table 2:** Differences in glucose levels between the carbohydrate and the fat-added test meal SD, standard deviation

Glucose measurement (mg/dl)	Measurement without the fat supplement (mean ± SD)	Measurement with the fat supplement (mean ± SD)	p-value
Fasting measurement	84.4±10	82.9±7.2	.767
Measurement 30 min	126.2±15	116.3±15	.059
Measurement 60 min	104.9±7	99±7	.028
Measurement 90 min	85.6±5	88.1±8	.218
Measurement 120 min	81.9±4	79.8±7	.254

Regarding Sigstad’s and Art’s score, a significant reduction was noted in late dumping symptoms after fat addition in the carbohydrate test meal, at the second assessment hour (p = 0.021), and in intensity of both early and late dumping symptoms, at the first and second assessment hour respectively (p = 0.007 and 0.012) (Table 3). 

**Table 3:** Differences in early and late dumping symptoms according to Sigstad’s and Art’s score between the two groups of the study. Q, questionnaire; SD, standard deviation

	Assessment without the fat supplement (mean ± SD)	Assessment with the fat supplement (mean ± SD)	p-value
Sigstad’s Q Score (1 hour)	9.9±6.7	6.6±5.3	.138
Sigstad’s Q Score (2 hour)	12.2±5.6	6.2±6	.021
Arts’s Q Score (1 hour)	8.5±4.6	4.2±3.6	.007
Arts’s Q Score (2 hour)	10±4.6	6.2±4.7	.012

## Discussion

The present study agrees with the findings of previous studies that after certain types of gastric surgery patients often develop postprandial symptoms related to DS [1,8,21,22,23]. In our case, 35% of the participants who had undergone gastric surgical procedures presented symptoms related to both early and late dumping. 

The pathophysiology of the dumping syndrome has not been completely elucidated. The central idea is that after reshaping the stomach and altering its volume capacity, the food does not necessarily have enough space to digest, and thus the unmanaged mass of food will move to the duodenal part of the small intestine. Normally, the stomach determines which parts of food are ready to transfer into the small intestine and releases them in small amounts via the pylorus, which allows the digested food to continue its course. The alteration of gastric anatomy and removal or bypass of the pylorus has an effect on gastric emptying and results to rapid delivery of large amounts of food into the duodenum [3,13].

As mentioned in the introduction section, the syndrome is distinguished in early and late dumping according to the symptoms it causes. In "early dumping", the symptoms start 15 to 30 minutes after eating a meal and include nausea, vomiting, bloating, cramps, diarrhea, dizziness and fatigue [24]. Hyperosmolar nutrients in the small bowel presumably cause a shift of fluid from the intravascular compartment to the intestinal lumen, resulting in a reduction in plasma volume, tachycardia, and rarely syncope. Furthermore, movement of fluid into the small bowel may cause distention and contribute to cramp like contractions, bloating and diarrhea [16]. Peptides, such as enteroglucagon, vasoactive intestinal peptide, peptide YY, pancreatic polypeptide, and neurotensin, are likely to be involved in this process [24]. On the other hand, late DS is characterized by symptoms that occur 1-3 h postprandially. Symptoms of late dumping consist of perspiration, faintness, decreased concentration, and altered levels of consciousness, among others. These symptoms are related to a reactive hypoglycemia that occurs 1-3 h postprandially [2]. Rapid delivery of undigested carbohydrates to the small intestine results in high glucose concentrations that induce a hyperinsulinemic response, resulting in subsequent hypoglycemia [16]. Also a study highlights the potential involvement of glucagon-like-peptide 1 in the pathogenesis of hypoglycemia accompanying the late DS [24].

It has been shown that the interaction of nutrients with the small intestine plays an important role in the regulation of gastric emptying and postprandial glycemia. Seems that when nutrients enter the small intestine, they generate feedback signals that slow gastric emptying and suppress appetite, via both neural and hormonal mechanisms [25]. Of the macronutrients, fat generates the most potent feedback, primarily because of its high caloric density and possibly because its absorption rate is relatively slower [26]. Several studies have tested the effect of fat addition to a carbohydrate meal, on gastric emptying and glycemic response. Gentilcore et al. [18] highlighted that adding 30ml of olive oil as a preload 30 minutes before a mashed potato meal markedly slows gastric emptying; delays postprandial rises in blood glucose, plasma insulin, and stimulates the excretion of glucagon-like peptide-1 (GLP-1) in type II diabetic patients. Moreover, in a study by Clegg et al. [19], the authors noted that adding different types of fat (sunflower oil, olive oil, butter or medium chain triglyceride oil) to a pancake meal containing 50gr of carbohydrate significantly reduced glycemic response and delayed gastric emptying in healthy volunteers. In the same study, the researchers also observed that monounsaturated and polyunsaturated fatty acids had a more potent effect on the above measured parameters than saturated fats. In our case although the glycemic response was generally reduced after fat addition during the second appointment, the reduction was modest and significant differences between the two groups were only noted at 60 mins after the ingestion of the meals. This finding can be attributed to the small sample of the study or/and the fact that the fat supplement was used in combination with the carbohydrate test meal and not independently a few minutes before, as done by previous studies [17,18] that observed greater reductions in blood glucose. 

With respect to the effect of fat ingestion on DS's early or late symptoms the current literature is limited. General dietary advice suggests that limiting refined carbohydrate-rich foods and increasing dietary fiber and complex carbohydrate foods can help prevent hypoglycemia. As far as proteins and fats are concerned, it has been recommended that they both elicit fewer symptoms and increased intake can help counterbalance possible negative caloric intake resulting from carbohydrate reduction or fear of eating [2,13]. However, when patients who had undergone Roux-en-Y gastric bypass were asked about which foods they avoid to prevent or relieve symptoms, they indicated fatty foods (55%) to a higher degree than sugar-rich foods (36%) [27]. In another recent study, the authors assessed symptoms associated with DS (Sigstad’s score), as well as glucose and insulin levels in a group of RYGB patients during a standardized liquid isocaloric meal challenge, containing either carbohydrate or a fat-rich meal [20]. Analysis of the collected data showed that the meal with predominant fat content resulted in as much perceived dumping symptoms as the carbohydrate profiled meal. As expected though, carbohydrate meal induced significantly higher rises in glucose and insulin levels than the fat meal. It is worth mentioning that the two previously mentioned studies assessed fatty and carbohydrate foods or test meals separately and not in combination, and this could explain the triggering of the symptoms by both. The present study tried to investigate the effect of the addition of fat to a carbohydrate meal on the appearance of dumping symptoms and glucose levels. As mentioned in the results section, a carbohydrate-based meal in combination with a moderate amount of fat significantly reduced both quantitatively and qualitatively dumping associated symptoms compared to carbohydrate alone. Furthermore, the fat supplement used in the present study contained mainly monounsaturated and polyunsaturated fatty acids which could also have played an important role in the alleviation of the symptoms. It has been shown by previous research that unsaturated fats increase satiety and the satiety hormone cholecystokinin (CCK) more than saturated fats [28] and that CCK is known to be an inhibitor of gastric emptying [29,30]. We acknowledge that the sample size of the present study was small and that its external validity can be potentially limited, but these are the preliminary results of the study which is still in progress and continues to recruit patients. Also, our findings can suggest directions for future research.

## Conclusions

In conclusion, incorporating unsaturated fat to a carbohydrate meal seems to attenuate postprandial rises in blood glucose and reduce the amount and intensity of dumping syndrome associated symptoms, in patients undergone gastric surgery. The study will continue to recruit participants in order to improve the sample size and reach more valid and concise conclusions.
